# Functional Outcomes of Distal Tibia Fractures Treated With Minimally Invasive Percutaneous Plate Osteosynthesis (MIPPO)

**DOI:** 10.7759/cureus.71669

**Published:** 2024-10-16

**Authors:** Muhammad Mannan, Shahzeen Eisha, Khandaker T Ahmed, Muhammad Ishfaq Mazari

**Affiliations:** 1 Trauma and Orthopaedics, Sheikh Zayed Medical College Rahim Yar Khan, Rahim Yar Khan, PAK; 2 Orthopaedic Surgery, University Hospitals Birmingham, Birmingham, GBR; 3 Trauma and Orthopaedics, Royal College of Surgeons in Ireland, Brighton, GBR; 4 Trauma and Orthopaedics, Birmingham Heartlands Hospital, Birmingham, GBR; 5 Orthopaedic Surgery, Sheikh Zayed Medical College Rahim Yar Khan, Rahim Yar Khan, PAK

**Keywords:** distal tibial fractures (dtf), external fixator, functional outcome, locking plate, mippo

## Abstract

Background: The tibia, a crucial long bone in the lower leg, plays a vital role in supporting mobility. Distal tibial fractures (DTFs) are relatively uncommon among lower extremity fractures. Minimally invasive percutaneous plate osteosynthesis (MIPPO) has become increasingly popular due to advancements in surgical techniques and its potential for positive clinical outcomes.

Objective: To evaluate the functional outcomes and complication rates of closed DTFs treated with MIPPO in adult patients, and to assess the impact of factors such as age, injury mechanism, and fracture classification on the treatment results.

Methodology: A retrospective study was conducted in the Department of Orthopedic Surgery at Sheikh Zayed Medical College Rahim Yar Khan, Pakistan from January 1, 2022, to December 31, 2022. Seventy patients, aged 18 to 60 years, were included. Postoperative follow-up assessments were conducted at six weeks, three months, and six months to evaluate functional outcomes.

Results: The majority of patients were male, with a mean age of 34 years. The most frequent cause of injury was road traffic accidents, followed by falls from height and other causes. Fractures were classified into three types: A1, A2, and A3. Functional outcomes at the final follow-up showed most patients had excellent to satisfactory results, while a smaller proportion had fair to poor outcomes. A few patients experienced postoperative complications, including malunion, infection, and ankle stiffness.

Conclusion: The study suggests that MIPPO is an effective surgical technique for managing DTFs, providing favourable functional outcomes with a relatively low complication rate. Therefore, MIPPO can be considered a valuable option in treating these fractures.

## Introduction

The tibia is a long bone located in the lower front region of the human leg. It is the body's strongest weight-bearing bone. Distal tibial fractures (DTFs) account for 10% of lower limb fractures, 85% of which also involve fibular fractures. These fractures can cause significant handicaps in individuals if not treated promptly and properly [1].

In terms of injury management, DTF presents a significant challenge to orthopaedic surgeons. The subcutaneous placement of the bone, combined with concomitant soft tissue damage, the high frequency of complex fractures, and poor vascularity often lead to delayed or non-union [2].

Previous research recommended a conservative approach for both extra-articular and intra-articular DTFs using a slab, followed by functional bracing or patellar tendon bearing. However, conservative management of DTF frequently results in a variety of problems, including malunion, non-union, and ankle stiffness, as well as a four to six-week period of cast application [3]. Most DTFs require surgical fixation, which necessitates meticulous pre-operative management. External fixators, interlocking nails, and locking plates are all options for fracture stabilization [4].

Due to the physiological properties of the distal tibia, including low blood supply and diminished muscle cover anteriorly, the most commonly reported complications are delayed or non-union, wound infection, and dehiscence. As a result, the best therapy for DTF in patients continues to be debatable. Because of its technological benefits and positive clinical outcomes, minimally invasive percutaneous plate osteosynthesis (MIPPO) has recently gained popularity [5].

MIPPO has been shown to overcome the challenges associated with other methods by preserving the vascularity of the fracture fragments and minimizing soft tissue damage [3]. Designed to prevent iatrogenic soft tissue injury and protect bone vascularity, MIPPO maintains the osteogenic fracture hematoma, which is critical for healing [6]. Additionally, the locking compression plate system secures screws to the plate, creating a stable, fixed-angle construct [7]. The locking screw-plate interface facilitates fracture repair without direct plate-bone contact, preserving the fracture hematoma and reducing the risk of non-union by maintaining microvascular circulation in the cortex and surrounding tissues [8]. This study aimed to evaluate the functional outcome of closed DTFs treated with MIPPO.

## Materials and methods

Study design

This retrospective study was conducted in the Department of Orthopedic Surgery at Sheikh Zayed Medical College Rahim Yar Khan, Rahim Yar Khan, Pakistan from January 1, 2022, to December 31, 2022. The sample size was calculated to be 70 patients using the WHO sample size calculator, based on a 95% confidence interval, a 7% margin of error, and an estimated 10% prevalence rate of DTFs [9]. Ethical approval was obtained from the Ethical Review Board (ERB) of Sheikh Zayed Medical College Rahim Yar Khan.

Inclusion and exclusion criteria

Patients of both genders, aged 18 to 60 years, with closed fractures or Gustilo-Anderson grade I open fractures of the distal tibia, as classified by the Arbeitsgemeinschaft für Osteosynthesefragen/Orthopaedic Trauma Association (AO/OTA) classification system, were included. Patients with type B and C fractures, Gustilo-Anderson grade II and III open fractures, multiple fractures, and comorbidities such as diabetes mellitus (DM) were excluded due to the increased risk of infection and other complications, which could skew the study’s results.

Data collection

The study involved a detailed review of clinical and radiological records of eligible patients. Evaluations were conducted in the orthopaedic outpatient department or emergency trauma ward, where anteroposterior (AP), lateral, and mortise images of the ankle were obtained for fracture identification. Initial stabilization of the ankle was achieved using a below-knee slab until definitive surgical intervention was performed.

All patients provided informed consent before participation, with assurance of no associated risk. The surgical procedure was performed under spinal anaesthesia. A vertical and curved incision was made at the level of the medial malleolus, preserving the fracture hematoma using a subcutaneous approach. Fracture reduction was assessed intraoperatively using fluoroscopy, and fixation was achieved using a locking compression plate (Synthes®, AO system; DePuy Synthes, Raynham, USA) and screws under C-arm guidance. Plates were positioned subcutaneously along the medial aspect of the tibia without disturbing the fracture hematoma. The incision was closed after removing the tourniquet.

Postoperative care

Postoperative care included intravenous antibiotics for three days. Sutures or staples were removed between the 12th and 14th postoperative days. A standardized rehabilitation protocol was followed, starting with non-weight-bearing mobilization on the fourth postoperative day. Active quadriceps exercises, including ankle and toe movements, began on the first postoperative day. Progressive weight-bearing was permitted based on radiographic evidence of fracture healing, and full weight-bearing was allowed only when sufficient healing was confirmed. Patient adherence to the rehabilitation protocol was monitored at each follow-up visit.

Follow-up and outcome assessment

Clinical and functional evaluations were conducted at six weeks, three months, and six months. Functional outcomes were assessed using the American Orthopaedic Foot & Ankle Society (AOFAS) score, and the radiographic union was evaluated by an independent orthopaedic surgeon not involved in the initial treatment.

Statistical analysis

Data were analyzed using Statistical Package for the Social Sciences (IBM SPSS Statistics for Windows, IBM Corp., Version 22.0, Armonk, NY). Quantitative variables, such as age and union time, were presented as mean ± standard deviation (SD). Qualitative variables, including gender, cause of injury, fracture classification, and functional outcomes, were expressed as frequencies and percentages. Stratification was performed to control for effect modifiers such as age, gender, and mechanism of injury. Post-stratification, chi-square tests were applied to categorical variables, and independent t-tests were used for continuous variables. A p-value ≤ 0.05 was considered statistically significant.

## Results

**Figure 1:**
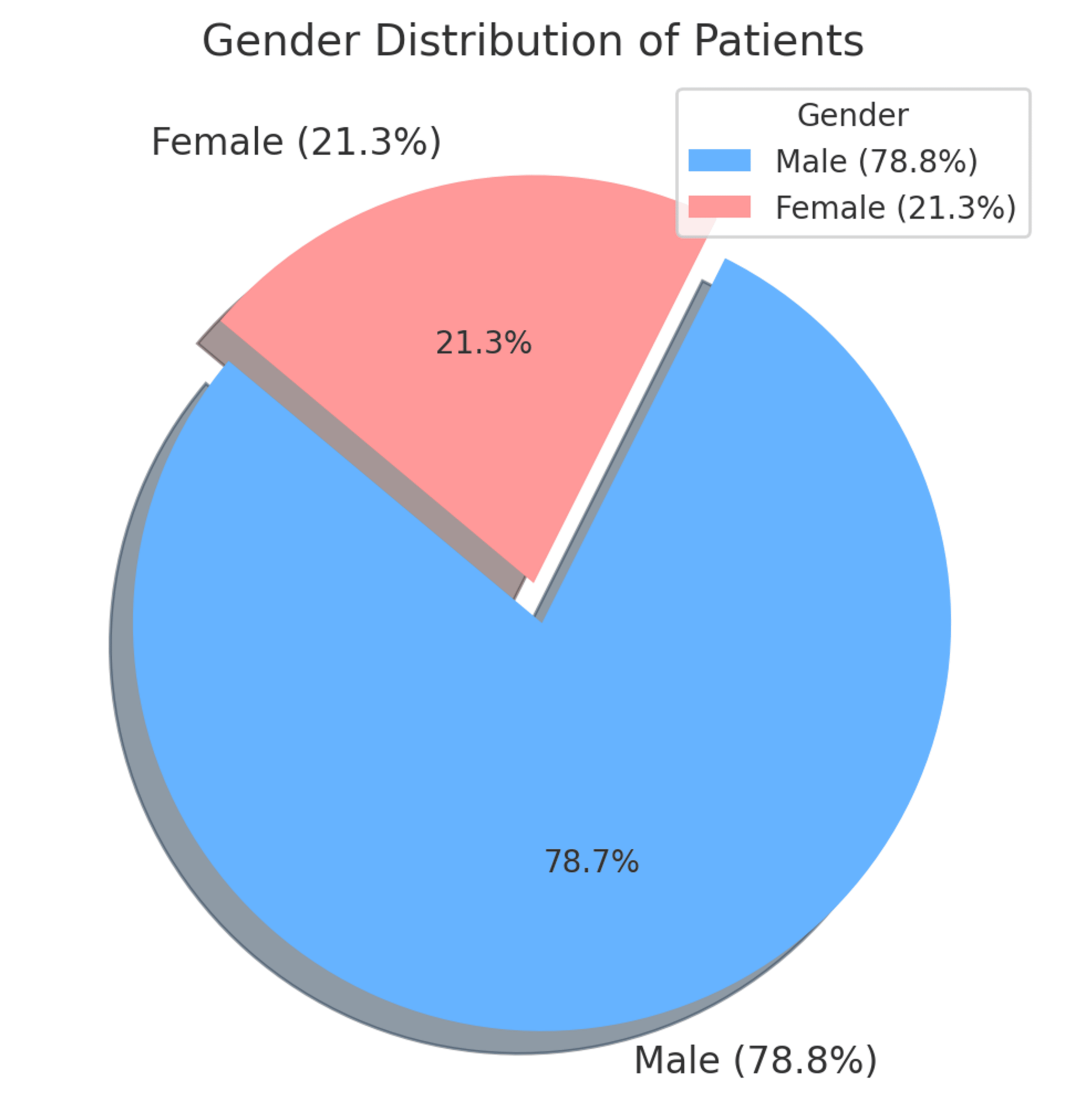
Gender Distribution of Patients

The study comprised a total of 70 patients. Results showed that 55 (78.8%) of the patients were male and 15 (21.3%) were female, as presented in Figure 1.

Table 1 provides a comprehensive summary of patient demographics, causes of injury, and fracture grading within the study population. The age distribution reveals that most patients were relatively young, with the majority falling into the 20-30 year age group, comprising 29 patients (41.4%). This was closely followed by the 31-40 year age group, which included 24 patients (34.3%). The 41-50 year age group made up 12 patients (17.1%), while those older than 50 years constituted the smallest group with only five patients (7.1%). The mean age across the study was 34.04 ± 9.66 years, indicating a predominantly younger population.

**Table 1:** Demographic Features of Patients RTA: road traffic accident

Category	Subcategory	Frequency	Percent (%)	Chi-Square Statistics	p-value
Age Groups	20-30 Years	29	41.4	11.83	0.008
	31-40 Years	24	34.3		
	41-50 Years	12	17.1		
	>50 Years	5	7.1		
Mean Age	Mean ± SD	34.04 ± 9.66			
Cause of Injury	Fall From Height	13	18.6	21.43	0.00002
	RTA	50	71.4		
	Other	7	10.0		
Grade of Fracture	A1	21	30.0	2.47	0.290
	A2	32	45.7		
	A3	17	24.3		
Total		70	100.0		

Regarding the causes of injury, road traffic accidents (RTAs) were found to be the leading cause, accounting for 50 cases (71.4%). Falls from height were the second most common cause, contributing to 13 cases (18.6%), while other causes made up the remaining seven cases (10%). This highlights RTAs as the predominant mechanism of injury in the study sample. In terms of fracture grading, A2 fractures were the most frequent, occurring in 32 patients (45.7%), followed by A1 fractures in 21 patients (30.0%), and A3 fractures in 17 patients (24.3%).

Statistical analysis using chi-square tests was conducted to explore potential associations between these variables. The chi-square test for age groups resulted in a statistic of 11.83 with a p-value of 0.008, indicating a statistically significant difference in the age distribution among patients (p < 0.05). Similarly, the test for causes of injury yielded a chi-square statistic of 21.43 with a p-value of 0.00002, suggesting a highly significant difference in the distribution of injury causes (p < 0.05). However, when examining the fracture grading, the chi-square statistic was 2.47 with a p-value of 0.290, indicating no statistically significant association between the fracture grades (p > 0.05). This suggests that while the distribution of patients by age and injury cause showed significant differences, the distribution of fracture grades was independent of these factors.

Table 2 summarizes the distribution of union times among the patients treated with the MIPPO technique. It shows that the highest percentage of patients, 29 (41.43%), achieved union within 12-16 weeks, followed by 22 (31.43%) who experienced union within 21-24 weeks. A smaller proportion, 19 (27.14%), had union times between 17 and 20 weeks.

**Table 2:** Union Time in Patients

Union Time	Frequency	Percent (%)	Chi-Square Statistics	p-value
12-16 weeks	29	41.43	-	-
17-20 weeks	19	27.14	-	-
21-24 weeks	22	31.43	-	-
Total	70	100.0	Chi-Square: 6.70	0.668

A chi-square test was conducted to examine if there was a statistically significant association between union time and other variables in the study (such as age, gender, or injury duration). The test produced a chi-square statistic of 6.70 with a p-value of 0.668. Since the p-value is greater than the conventional threshold of 0.05, we conclude that there is no significant association between the variables and the union time distribution.

Table 3 presents the functional outcomes of patients treated with the MIPPO technique. Out of the 70 patients, 21 (30%) had an excellent outcome, while the majority, 37 (52.9%), had satisfactory results. Nine (12.9%) experienced a fair outcome, and only three (4.3%) had poor outcomes.

**Table 3:** Functional Outcomes After Bridge Plating

Functional Outcome	Frequency	Percent (%)	Age Groups	Gender	Mechanism of Injury	Chi-Square Statistics	p-value
Excellent	21	30.0	-	-	-	-	-
Satisfactory	37	52.9	-	-	-	-	-
Fair	9	12.9	-	-	-	-	-
Poor	3	4.3	-	-	-	-	-
Total	70	100.0	Included	Included	Included	6.70	0.668

The total chi-square statistic is 6.70 with a p-value of 0.668, which is greater than the significance threshold of 0.05. This indicates that there is no statistically significant association between the functional outcomes and the factors considered (age groups, gender, and mechanism of injury). Therefore, variations in functional outcomes appear to be independent of these factors in this study.

In our study, the majority of patients, 61 (87.1%), did not experience any postoperative complications. Among those who did, the most common complication was malunion in four (5.7%) patients, followed by infection in three (4.3%) patients, and ankle stiffness in two (2.9%) patients.

A chi-square test was conducted to explore the potential association between postoperative complications and variables such as age, gender, and injury duration. The test yielded a chi-square statistic of 6.70 with a p-value of 0.668. Since the p-value is greater than 0.05, this result suggests that there is no statistically significant association between the occurrence of complications and the factors considered (Table 4).

**Table 4:** Postoperative Complications

Complication Type	Frequency	Percent (%)	Chi-Square Statistics	p-value
None	61	87.1	-	-
Malunion	4	5.7	-	-
Infection	3	4.3	-	-
Ankle Stiffness	2	2.9	-	-
Total	70	100.0	Chi-Square: 6.70	0.668

## Discussion

DTFs are particularly challenging to treat due to the vulnerable condition of the surrounding soft tissue and the degree of impaction at the time of injury, both of which play a significant role in determining long-term clinical outcomes. The primary objective of operative therapy is to achieve anatomical alignment of the joint surface while maintaining adequate stability to allow for early mobility. This is achieved using techniques that minimize bone and soft tissue devascularization, thereby reducing the risk of treatment-related complications [10]. The present study sought to evaluate the functional outcomes of 70 patients managed with the MIPPO technique for DTFs.

Our results showed that 78.8% of the patients were male and 21.3% were female, aligning with the findings of other national and international studies. This distribution is illustrated in Figure 1. For instance, Illur et al. [10] reported a gender distribution of 65% males and 35% females in a study from India, while another Indian study reported 80% males [2]. These findings are consistent with similar studies [11,12]. In a Pakistani study, 68.6% of the patients were male, and 31.4% were female [1]. Such gender distributions reflect the global trend of a higher incidence of DTFs in males, which is often attributed to more frequent exposure to high-energy trauma, such as RTAs.

The mean age of patients in this study was 34.04 ± 9.66 years, closely comparable to the mean ages reported in other research [13-15]. For example, Marei et al. [16] reported a mean age of 40.16 ± 11.62 years in a study conducted in Egypt. In terms of the causes of injury, RTAs were the most common, accounting for 71.4% of cases, followed by falls from height (18.6%) and other causes (10%). This distribution of injury mechanisms is consistent with other studies on DTFs [17], highlighting the need for early intervention in high-energy trauma cases. The fractures were classified as A1 in 30% of patients, A2 in 45.7%, and A3 in 24.3%, aligning with patterns observed in the literature [17].

In terms of functional outcomes, 30% of patients achieved excellent results, 52.9% had satisfactory outcomes, 12.9% had fair outcomes, and 4.3% had poor outcomes. These results underscore the efficacy of the MIPPO technique in achieving positive outcomes for the majority of patients. Postoperative complications were observed in 12.86% of patients, including malunion in 5.7%, infection in 4.3%, and ankle stiffness in 2.9%. These complication rates are consistent with those reported in other studies [13-17], reinforcing the notion that the MIPPO technique provides a reliable treatment option with relatively low complication rates. In a related study, delayed union was identified as the most frequent complication following the MIPPO procedure for DTFs, followed by malunion (6.67%), infection, and implant failure (3.33%) [18].

Our findings align closely with those of Paluvadi et al. [15], who similarly observed favourable outcomes with the MIPPO technique. Furthermore, when comparing MIPPO to intramedullary nailing, Liu et al. [19] found that MIPPO resulted in lower rates of malunion and fewer wound complications. These findings highlight the advantages of using MIPPO, particularly in minimizing soft tissue disruption and promoting faster recovery with fewer complications compared to traditional open surgery or intramedullary nailing.

Limitations

This study has several limitations that should be considered. One major limitation is the relatively small sample size, which may reduce the generalizability of the findings. A larger, multi-centre study involving a more diverse patient population would be necessary to validate these results and provide a more comprehensive understanding of the functional outcomes associated with MIPPO in DTFs. Additionally, conducting the study in a single hospital setting may have introduced Berksonian bias, as the hospital patient population may not represent the broader community. Patients treated in different healthcare environments, such as outpatient clinics or rural settings, may exhibit different characteristics and outcomes, limiting the ability to generalize our findings to a wider population.

Another limitation is the retrospective nature of the study, which inherently carries risks such as potential inaccuracies in recorded data and the absence of randomization. These factors can affect the reliability of the results. Furthermore, the variability in factors like surgical expertise, postoperative care, and patient adherence to rehabilitation protocols was not controlled, which could have influenced the functional outcomes observed in this study.

## Conclusions

In summary, the study highlights that MIPPO is an effective and minimally invasive approach for managing DTFs, offering favourable functional outcomes for the majority of patients. The technique's ability to preserve soft tissue and vascular supply significantly reduces the risk of complications, such as malunion and infection, and supports early mobilization, which is crucial for optimal recovery. Additionally, the low incidence of poor outcomes in this study underscores the reliability of MIPPO as a preferred treatment option. These findings reinforce the value of MIPPO as a reliable option for orthopaedic surgeons, particularly in complex cases where other fixation methods may present greater risks or challenges. Overall, MIPPO facilitates quicker rehabilitation and improves patient quality of life post-surgery.
